# Practice parameters for the use of actigraphy in the military operational context: the Walter Reed Army Institute of Research Operational Research Kit-Actigraphy (WORK-A)

**DOI:** 10.1186/s40779-020-00255-7

**Published:** 2020-05-31

**Authors:** Jaime K. Devine, Jake Choynowski, Tina Burke, Kajsa Carlsson, Vincent F. Capaldi, Ashlee B. McKeon, Walter J. Sowden

**Affiliations:** 1 Operational Fatigue and Performance, Institutes for Behavior Resources, Baltimore, MD 21218 USA; 2 Behavioral Biology Branch, Walter Reed Army Institute of Research, 503 Robert Grant Ave, Silver Spring, MD 20910 USA; 3 Tripler Army Medical Center, Honolulu, HI 96859 USA

**Keywords:** Actigraphy, Sleep-wake patterns, Military sleep assessment, Operational environment, Scoring methodology

## Abstract

**Background:**

The Walter Reed Army Institute of Research (WRAIR) Operational Research Kit-Actigraphy (WORK-A) is a set of unique practice parameters and actigraphy-derived measures for the analysis of operational military sleep patterns. The WORK-A draws on best practices from the literature and comprises 15 additional descriptive variables. Here, we demonstrate the WORK-A with a sample of United States Army Reserve Officers’ Training Corps (ROTC) cadets (*n* = 286) during a month-long capstone pre-commissioning training exercise.

**Methods:**

The sleep of ROTC cadets (*n* = 286) was measured by Philips Actiwatch devices during the 31-day training exercise. The preliminary effectiveness of the WORK-A was tested by comparing differences in sleep measures collected by Actiwatches as calculated by Philips Actiware software against WORK-A-determined sleep measures and self-report sleep collected from a subset of ROTC cadets (*n* = 140).

**Results:**

Actiware sleep summary statistics were significantly different from WORK-A measures and self-report sleep (all *P* ≤ 0.001). Bedtimes and waketimes as determined by WORK-A major sleep intervals showed the best agreement with self-report bedtime (22:21 ± 1:30 vs. 22:13 ± 0:40, *P* = 0.21) and waketime (04:30 ± 2:17 vs. 04:31 ± 0:47, *P* = 0.68). Though still significantly different, the discrepancy was smaller between the WORK-A measure of time in bed (TIB) for major sleep intervals (352 ± 29 min) and self-report nightly sleep duration (337 ± 57 min, *P* = 0.006) than that between the WORK-A major TIB and Actiware TIB (177 ± 42, *P* ≤ 0.001).

**Conclusions:**

Default actigraphy methods are not the most accurate methods for characterizing soldier sleep, but reliable methods for characterizing operational sleep patterns is a necessary first step in developing strategies to improve soldier readiness. The WORK-A addresses this knowledge gap by providing practice parameters and a robust variety of measures with which to profile sleep behavior in service members.

## Background

Service members are more likely than civilian populations to experience sleep disturbances, including shortened sleep, fragmented sleep, and difficulty falling/maintaining asleep [1–4]. In many ways, these sleep disturbances may closely resemble those seen in shift workers and other operational contexts [5–7]. However, industrial operations have greater capacity to control or document work schedules [8], whereas conditions during military operations are unpredictable [9]. Given these differences in sleep patterns, sleep in the military operational context should be quantified differently than how it is in civilians.

To accurately profile the uniquely erratic sleep patterns of soldiers in the military operational context, researchers on the Operational Research Team (ORT) at the Walter Reed Army Institute of Research (WRAIR) have developed the WRAIR Operational Research Kit-Actigraphy (WORK-A). The WORK-A is a set of actigraphy-derived measures adapted from commercial industry standards for the analysis of sleep patterns that are specific to active-duty soldiers in operational domains.

### Actigraphy

Actigraphy has been a mainstay of sleep research since the late 1970s [10] and been used for the analysis of circadian and rest-activity rhythms (RAR) in healthy and specialty populations [11–16]. Specifically, actigraph devices detect body movement and sum these movements into digital activity counts across a predetermined epoch interval. Actigraphy software infers sleep-wake patterns from activity counts using computerized scoring algorithms that have been validated against polysomnography (PSG). However, while PSG (the gold standard for the measurement of sleep) uses standardized rules for scoring sleep [17], there is no currently established consensus for scoring actigraphy [18, 19]. Even within actigraphy platforms validated against PSG, such as Philips Actiware [20–22], the quality control of sleep data is left to the interpretation of the researcher.

Supplementary data can help researchers differentiate actual sleep from periods of inactivity or removal of the device. Research actigraphy devices have begun to include additional features such as recording exposure to light, measuring heart rate and temperature, and providing options for the wearer to manually record an event such as bedtime. Newer models can also sense when the device is not being worn and can alert the user to put the watch back on. Apart from the device, research participants will frequently complete self-report diaries during the data collection period which helps add context to the objective sleep/wake schedule of the individual of the individual. However, these supplementary measures may not always be practical, as they increase the burden of data collection on the participant. Characterizing sleep in populations with demanding and irregular schedules may have to rely on stand-alone analysis of actigraphy data without context. Reliable methods of analysis must therefore be established to profile sleep in specific populations, such as active duty military personnel.

Philips default actigraphy settings record one interval of sleep lasting longer than 3 h per 24 h period (also called a major sleep interval) and compute average sleep measures (total sleep time [TST], sleep efficiency [SE], wake after sleep onset [WASO], etc.) collected across all sleep intervals. Researchers can adjust the settings to account for minor sleep intervals (between 15 min to 3 h in length) and to record more than one sleep interval per day.

However, sleep measures are averaged across all collected sleep intervals without a clear distinction between major and minor sleep intervals. Averaging major and minor sleep intervals together can result in a shorter average sleep duration, even though the individual is receiving more sleep within a 24-h period by taking extra sleep opportunities (i.e., naps). Moreover, measures of sleep quality, such as SE or WASO, are calculated within the bounds of a single sleep interval and thus do not account for fragmentation, due to sleep being split into multiple intervals across the day. It is important to understand how frequently within a 24-h period an individual is sleeping and the length of each sleep interval to accurately estimate average sleep duration and quality.

Standardization of research procedures in actigraphy is progressing. Several critiques and guidelines have been published outlining appropriate actigraphy procedures over the years [18, 23–27]. Moreover, the methodological issues inherent to actigraphy have been addressed for specific populations, such as individuals with sleep disorders [26], nurses [28], cancer patients [19], mothers and infants [29], dementia patients [30] and individuals with traumatic brain injuries [31]. The Society of Behavioral Sleep Medicine and The Journal of Clinical Sleep Medicine recently produced comprehensive instructional manuals for behavioral sleep assessment using actigraphy in general populations [32] and clinical practice [33].

### Walter reed Army Institute of research operational research kit-Actigraphy (WORK-A)

The WORK-A aims to address the problems interpreting actigraphy data outlined above in a population that has not previously been addressed, i.e., military personnel. The primary goal of the WORK-A is to accurately profile soldier sleep rather than rest-activity or circadian rhythm. As such, only measures related to sleep duration and sleep quality or methods to facilitate interpretation of these data were considered for incorporation into the kit. The selection for measures to include in this kit was informed by data collected from service member populations as part of the ORT’s operational field studies as well as input from sleep research professionals and U.S. Army officers. The WORK-A is designed to apply to a wide range of operational scenarios, but for the purposes of this paper, it has been applied to Reserve Officers’ Training Corps (ROTC) cadets undergoing Advanced Camp.

The Cadet Summer Training Advanced Camp at Fort Knox is a 31-day training event designed to assess an ROTC cadets’ ability to meet US Army standards through a tiered training structure using light infantry tactics as the instructional medium. ROTC Advanced Camp is the U.S. Army’s largest training exercise and the U.S. Army Cadet Command’s capstone training event. ROTC Advanced Camp provides a realistic glimpse into the military operational context, thus making it an ideal setting for the initial application of the WORK-A.

Here, we present the methodology for actigraph configuration and pre-processing, the minimum data requirements, and the calculation of variables from processed and exported actigraphy data, which constitute the full research toolkit known as the WORK-A. The toolkit relies on methods that are established in the literature and attempts to minimize novel methodologies or subjective interpretation. To maximize the measuring accuracy of soldier sleep, the WORK-A has been applied to a dataset of activity recordings collected from ROTC cadets undergoing Advanced Camp to assess agreement between traditional measure of actigraphy, the WORK-A and self-report measures of sleep using Bland-Altman analysis [34, 35], Student’s *t*-tests and descriptive statistics.

## Methods

### Selection of measured parameters

The first step in developing the WORK-A was a literature review to determine the most applicable practice parameters for actigraphy, methods for sleep/wake determination for populations with unpredictable schedules, and measures for quantifying sleep. The literature review was conducted using PubMed and Google Scholar search engines using the following combinations of search terms: 1) actigraphy or accelerometry; 2) practice parameters, methods, methodology or practices; and 3) sleep, sleep medicine, sleep research, sleep patterns or sleep/wake behavior. Additional searches were conducted to identify articles applying actigraphy to specific populations that may share common methodological issues with US Army soldiers, such as individuals in specific operational contexts (shift workers, pilots, military, doctors and nurses, long-haul drivers, athletes), individuals with atypical rest-activity rhythms (sleep disordered individuals, circadian disordered patients, parents of infants, etc.), patient populations with comorbid sleep issues (cancer, dementia, mental health disorders) or non-industrial indigenous groups. Practices and methods from areas outside sleep research/sleep medicine (i.e., circadian misalignment, fragmentation index [36], intradaily variability, interdaily stability, light exposure, RAR, etc.) were not incorporated into the WORK-A. Moreover, not yet validated methods of sleep/wake determination from sleep research were not considered.

While the literature still recommends the use of sleep diaries or other supplementary data to hand score actigraphy, there is reliable confidence in the ability of validated algorithms to accurately measure habitual sleep patterns [19, 26, 27, 32, 33]. By relying on automated scoring algorithms from validated devices with supporting software, the WORK-A can more easily be reproduced by fellow researchers. The literature review also revealed key variables for the reporting of habitual sleep patterns, specifically, measures of habitual bedtime and waketime, time in bed (TIB), sleep onset latency (SOL), TST, WASO and SE [19, 27, 32].

### Addressing gaps between sleep measures for civilian versus military populations

The goal of the WORK-A is not only to quantify military sleep patterns but also to identify how sleep patterns may contribute to military readiness. However, the military operational domain is a variable environment, and soldier sleep patterns can differ greatly based on the context of the mission. Aspects of sleep that may correlate with performance in one population of soldiers (for example, a tank crew) cannot be assumed to be generalizable to a different population (such as Army Rangers). To address this variability, the WORK-A quantifies sleep via the key sleep variables described above (bedtime, wake time, TIB, SOL, TST, WASO, and SE) for specific types of sleep (specifically, all sleep intervals, minor sleep intervals, major sleep intervals and daily sleep) to create a versatile toolkit from which different variables can be selected to address research questions relating to a wide range of soldier groups.

### Actigraphy measures in WORK-A

Key actigraphy variables and the WORK-A measures are defined in Table 1. The WORK-A is designed to be adaptable for use with any actigraphy device with adjustments as necessary. Philips Actiware and Actiwatch devices (Actiwatch 2.0, Philips Respironics, Murrysville, PA) were used for the purposes of this paper. Using default settings, Philips Actiware averages key sleep variables (TIB, SOL, TST, WASO, SE) across all recorded sleep intervals to provide a summary statistic. Philips Actiware also provides a mobility measure called the fragmentation index, which is defined as the “sum of percent mobile and percent immobile bouts less than 1 minute in duration to the number of immobile bouts for a given interval”. This measure has been applied to sleep intervals to measure sleep fragmentation [37, 38], but it has not been sufficiently utilized in the sleep literature to be considered a key sleep variable [19, 27, 32]. The WORK-A investigated key sleep variables averaged over all sleep intervals, as well as major sleep intervals only, minor sleep intervals only, and overall daily sleep. Daily sleep measures are calculated by combining all sleep intervals from which an individual woke within a 24-h period into a sum for that day. For example, if on a given day, an individual slept for 5 h and then took a 90-min nap, their daily TST would be 6.5 h. Twenty-four-hour periods begin at 12:00 (noon) and end at noon the subsequent day, as per Philips Actiware specifications. Average and median bedtime and wake time, as well as sleep midpoint, were also calculated.

**Table 1:** Key actigraphy variables and actigraphy measures in WORK-A

Metric types	Measure	Definition
Key actigraphy variables	Time in bed (TIB)	The average time elapsed between the start time and the end time of a given rest interval (representing time spent in bed/at rest/attempting to sleep) in minutes
	Total sleep time (TST)	The total number of minutes within a given rest interval that have been scored as sleep by the sleep interval (representing time when sleep is occurring) detection algorithm
	Sleep onset latency (SOL)	The time required for sleep to start after initiating the intent to sleep. The time, in minutes, between the start of a given rest interval and the onset of sleep as scored by the sleep interval detection algorithm
	Wake after sleep onset (WASO)	The total number of minutes scored as wake within a given sleep interval
	Sleep efficiency (SE)	The percentage of time spent in bed sleeping. Scored as TST/TIB multiplied by 100
Actigraphy measures in Walter Reed Army Institute of Research Operational Research Kit-Actigraphy (WORK-A)	All intervals actigraphy measures	TIB, TST, SOL, WASO and SE averaged across all sleep intervals
	Major interval actigraphy measures	TIB, TST, SOL, WASO and SE averaged across only major sleep intervals (sleep intervals lasting longer than 3 h)
	Minor interval actigraphy measures	TIB, TST, SOL, WASO and SE averaged across only minor sleep intervals (sleep intervals lasting between 15 min and 3 h)
	Daily actigraphy measures	TIB, TST, SOL, WASO and SE combined for all sleep intervals within a 24-h period, then averaged across days worn
	Sleep midpoint	Halfway point between the time of sleep onset and final awakening
	Time worn	Number of minutes or days that device was worn
	Daily sleep intervals (DSI)	The average number of sleep intervals recorded across all days worn
	DSI bedtime (s)	Average start time for a sleep interval by DSI number within a 24-h period
	DSI waketime (s)	Average end time for a sleep interval by DSI number within a 24-h period
	Soldier sleep below minimum (SSBM)	Number of days during wear that participant slept less than a researcher-specified number of hours

The WORK-A also took into account the time worn and calculates the number of times within a 24-h period (defined as 12:00–12:00) sleep occurs to determine daily sleep intervals (DSI) to account for fragmentation of sleep opportunities. The number of sleep intervals per 24-h period was derived from the DSI based on interval start time. The average bedtime and wake time for the first and second DSI occurring within a day were also included in the WORK-A. Time worn was calculated by summing the number of calendar days during the study period on which the participant wore the watch, subtracting off-wrist days or excluded periods. This ensures that days on which no sleep occurred are still accounted for in the data. Next, we averaged the number of sleep intervals per day, start time and end time of intervals (bedtime and waketime) and the length of intervals across the total days the participant wore the actigraph. By doing this, we can more thoroughly describe when and how well soldiers are maximizing their opportunities for sleep.

The final variable in the WORK-A quantifies the soldier as above or below a specified minimum. The National Sleep Foundation recommends 7 or more hours of sleep per night for healthy functioning [39], and while there is currently no Army-mandated minimum sleep requirement [40], the Army has previously recommended that soldiers receive a minimum of 4 h of sleep within a 24-h period [41]. The soldier sleep below minimum (SSBM) variable can be adjusted to specify a threshold for the minimum acceptable level of sleep (specified for these analyses as daily TST less than 4 h) by summating a series of conditional Boolean arguments that categorize daily TST as over (false = 0) or under (true = 1) the designated threshold during a specified time period. With this, we can then determine how many days during the study period soldiers fail to meet a specified minimum recommendation for sleep duration, even if they habitually meet the minimum.

The WORK-A also has two additional features to help researchers conceptualize habitual sleep patterns across 24 h. These features calculate a separate dataset with hourly bins of 1) activity counts and 2) the likelihood of a sleep interval occurring during that hour and compiles this information by hour (0000–2300) for each participant within a study sample in a separate .csv file. These features are not validated measures but can be used to visualize activity patterns across the day.

### Pre-processing of the actigraphy data

Actigraphy data are retrieved from devices and saved in a common software database. Sleep intervals are then automatically determined by the built-in actigraphy software platform of the device. Computerized scoring of actigraphy data collected on Philips Respironics Actiwatch models is therefore based on Philips Actiware settings for automatically determining major and minor rest intervals with medium sensitivity (Actiware 6.0.9, Philips Respironics, Murrysville, PA). More than one sleep interval is permitted per day. Philip’s default setting for the determination of sleep onset is a period of 10 or more minutes wherein the activity count (AC) is below the threshold of immobility (≤40 counts/epoch). The minimum permitted duration for a sleep interval was 15 min, in contrast to the Philips default recommendation of 40 min. There was no set maximum duration for sleep interval duration.

All data were cleaned by one trained researcher who records any adjustments in a standardized cleaning log. A second trained researcher performed a data quality control check on 10% of the total dataset that is randomly selected to ensure reliability in data cleaning. At a minimum, 72 consecutive hours of data collection with data binned in ≤1-min epochs was required for viable sleep-wake data collected by wrist actigraphy. Days (i.e., 24-h periods) with missing or unreadable data for 4 or more hours were excluded from analysis. Recordings of activity prior to the study start date or after the study end date were excluded from the analysis as well. In instances where actigraphy data was supplemented by sleep diaries or other daily schedule information, the researchers adjusted sleep and wake times in accordance with that information on a study-by-study basis, depending on the source and dependability of the supplementary data. For stand-alone actigraphy data analysis, sleep intervals were only reset in instances when the algorithm missed an instance of wake onset (defined as ≥10 min where AC ≥ 40) or sleep onset (defined as ≥10 min where AC ≤ 40).

### Off-wrist determination via the WORK-A transitional dynamics

The distinction between periods of inactivity due to sedentary behavior versus removal of the device is determined by capacitive sensor automatic off-wrist detection when available. In devices that lack capacitive sensors (i.e., Philips Actiwatch 2.0), off-wrist time is defined by a transitional dynamic approach. Instances where there is an interval of no or little activity (AC = 0 during the interval in question or 0 < AC < 10% of the interval duration) but activity is consistently high prior to and following the period of inactivity (≥ 10 min where AC ≥ 40 at least 50% of epochs) are considered off-wrist. Shorter periods of inactivity that are not coded as a sleep interval do not affect data analysis of sleep measures. Hence, for the purposes of sleep-wake determination, periods of off-wrist inactivity are excluded only if the interval has been automatically scored as a sleep interval by the Philips algorithm or if the interval is over 4 h in length.

### Rectifying misrepresentative averages with the WORK-A

Philips Actiware allows for the export of all interval data (daily intervals, active intervals, rest intervals and sleep intervals) but importantly, key sleep variables (TIB, SOL, TST, WASO, SE) are only computed for sleep intervals. Data can be exported for each individual interval, but there is also the option to export summary statistics averaged across all available intervals. The resulting measure of sleep quantity or quality does not take into account how the number of recorded sleep intervals may differ from the number of days that the device was worn though.

Philips Actiware infers the statistics of bedtime, waketime or TIB from interval start time, end time and duration, respectively. The sleep summary statistics do not calculate average start or end times, and sleep interval duration does not account for time in bed prior to falling asleep (i.e., SOL) or time spent in bed after final awakening (called Snoozetime in Actiware). These measures must therefore be added to the sleep interval duration measure to more accurately estimate TIB.

Another issue is the averaging of time to determine habitual bed- or waketime. Actiware defines a day from 12:00–12:00 (noon to noon) rather than 00:00–00:00 (midnight to midnight). While defining a day at midnight is impractical for the purposes of quantifying sleep, defining a day as noon to noon is also not ideal. If a 12-h clock is used, it may be difficult to determine whether time measures refer to AM or PM. A 24-h clock, in contrast, resets at midnight (i.e., 00:00). While it is commonly understood that 00:03 on 1/2/2019 is later than 23:52 on 1/1/2019, averaging these two times mathematically yields a habitual bedtime of 12:37, indicating that the participant normally went to sleep just after noon. For individuals with normal sleep schedules, these errors may be obvious and easy for researchers to catch, but they could skew the interpretation of sleep behavior in service members in an operational context. The WORK-A attempts to resolve this issue in two ways. First, prior to calculating averages, the exact time was transformed into a 12 (noon) to 36 (subsequent noon) hour clock by adding 24 h to each hour between midnight and subsequent noon. In the context of the previous example, 00:03 would be recalculated as 24:03, which is numerically greater than 23:52 and yields a more accurate average (23:57 vs. 12:37). Times can be translated back into 24 h time by subtracting 24:00 from values occurring between midnight (24:00) and subsequent noon (36:00). Second, the WORK-A provides not only average bed- and waketimes but also median bed- and waketimes to provide a measure of habitual sleep schedule, which may be more resilient to outliers.

### Comparison of default Actiware summary statistics to those of the WORK-A in ROTC advanced camp cadets

ROTC cadets during Advanced Camp serve as an ideal candidate group to demonstrate the WORK-A due to the extended duration of the study period (31 days), the structured design of camp that enabled testing cadets across a range of mission scenarios in a controlled environment, and the ability to collect self-report data of sleep quality from the cadets. This study was approved by the Walter Reed Army Institute of Research Human Use Review Committee, protocol #2532C, and was performed in accordance with the ethical standards of the 1964 Declaration of Helsinki. This study was a part of a larger experiment aimed at assessing sleep patterns and sleep quality and their relationships with health and performance. Participants were recruited from cadets undergoing Advanced Camp during routine in-processing on the first day of camp and gave consent separately for the actigraphy and survey portions of the study. Participants were asked to wear a wrist actigraph (Actiwatch 2.0 Philips Respironics, Murrysville, PA) continuously throughout camp (up to 31 days). Activity data were collected in 30-s epochs, and sleep-wake determination was computed by the Actiware 6.02 scoring algorithm. Participants were also asked to complete the Pittsburgh Sleep Quality Index (PSQI), a one-time self-report measure of sleep quality from the past month at the end of camp [42].

Summary statistics for key sleep variables were batch exported from Actiware 6.02 prior to WORK-A pre-processing to serve as traditional actigraphy measures of sleep. Sleep-wake determination by the Actiware 6.02 scoring algorithm was not modified by researchers for this traditional actigraphy dataset. Trained researchers then pre-processed each participant’s actigraphy data according to the WORK-A standards described above. Cleaned actigraphy data were exported as individual .csv files and compiled using WORK-A computational methods as described above.

### Statistical analysis

Descriptive summary statistics for the traditional and WORK-A actigraphy datasets were computed using Excel 2013 (Microsoft Corp, USA) and SPSS version 25 (IBM Corp, USA). Paired samples *t*-tests were run to compare differences in target sleep variables that existed in both the Actiware sleep summary statistics and the WORK-A. Mean difference scores were computed between habitual bedtime, waketime and sleep duration during camp as recorded by the self-report PSQI, the Actiware summary statistics TST and TIB, and the WORK-A measures TST and TIB. Single sample *t*-tests were conducted to determine if a statistically significant difference existed between mean difference scores. Bland-Altman plots further examined the mean difference between measures of sleep using Excel 2013 and SPSS version 25.

## Results

### Profiling soldier sleep in ROTC cadets via the WORK-A

Actigraphy data were collected from 286 ROTC Advanced Camp cadets (186 men, 68 women and gender unavailable for 32; average age: 22 ± 3 years*,*). Cadets wore the watch for an average of 22 ± 7 days during the 31 days of camp. Actiwatches were off-wrist for an average of 0.65 ± 1.38 days between the time that the cadets first put on and when they last removed the watch. Cadets slept between once and twice per day (DSI: 1.45 ± 0.25). The median first DSI bedtime was 21:12 ± 1:29 h, and the first DSI wake time was 01:47 ± 1:43 h. The median second DSI bedtime was 01:50 ± 2:02 h, and the second DSI wake time was 04:50 ± 1:51 h.

**Fig. 1:**
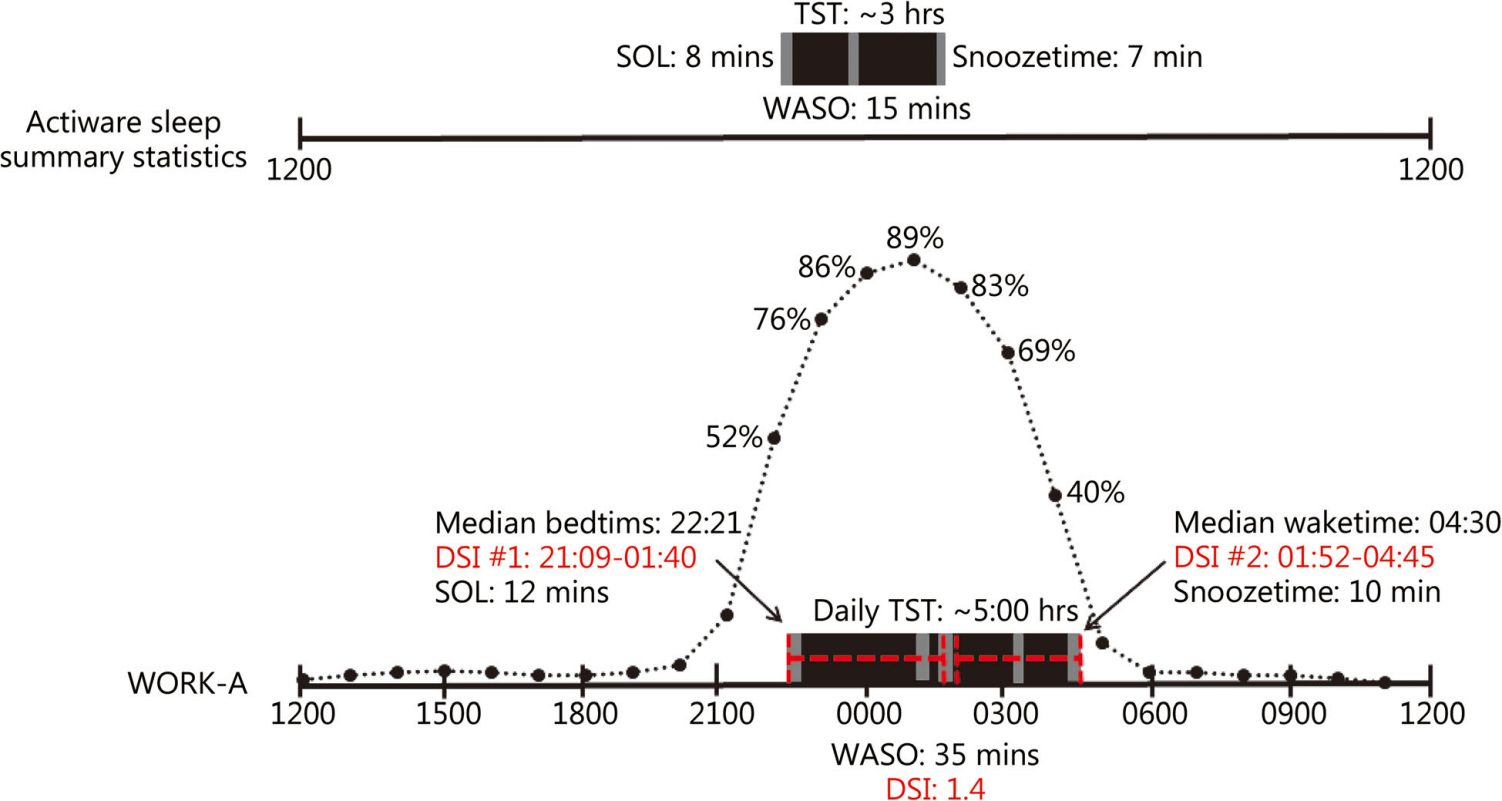
Visualization of an average day for ROTC cadets during advanced camp using Actiware sleep summary statistics vs. the WORK-A toolkit. Bed- and waketimes during Advanced Camp based on measures extracted from either Actiware Sleep Summary Statistics (top) or the WORK-A (bottom). The cadet’s sleep duration (TST:), sleep quality (SOL, WASO and Snoozetime:), median bed- and waketimes, TST, SOL, WASO, and snooze time from the WORK-A major interval subset are depicted, as the major intervals show the most agreement with the PSQI self-report. The hourly percent likelihood of sleep occurrence is represented by the dashed black line (). Daily sleep interval (DSI) information is represented in red

The results for comparable key sleep variables as measured by Actiware sleep summary statistics and WORK-A measures are summarized in Table 2. The Actiware sleep summary statistics were significantly different from all comparable WORK-A measures (all *P* ≤ 0.001). Key sleep variables were significantly different between the WORK-A all intervals, major, minor and daily (all *P* ≤ 0.05) variables except for the WORK-A major and daily measures of WASO (*t* = 0.55, *P* = 0.58). There were no strong correlations (*r* > 0.5) between any WORK-A measures and/or Actiware summary statistics. Estimated average sleep profiles as determined by summary statistics versus the WORK-A are visualized in Fig. 1.

**Table 2:** Results for key sleep variables as measured by Philips Actiware and the WORK-A

Sleep parameters	Actiware sleep summary statistics	WORK-A all intervals averages	WORK-A daily averages	WORK-A major averages	WORK-A minor averages
Time in Bed (TIB; min)	177 ± 42*	259 ± 48	366 ± 44	352 ± 29	53 ± 19
Total sleep time (TST; min)	161 ± 39*	214 ± 44	303 ± 44	293 ± 33	38 ± 16
Sleep onset latency (SOL; min)	8 ± 4*	10 ± 7	14 ± 10	12 ± 9	6 ± 4
Wake after sleep onset (WASO; min)	15 ± 6*	25 ± 8	36 ± 10	35 ± 15	4 ± 5
Sleep efficiency (SE; %)	81 ± 4*	80 ± 6	83 ± 5	83 ± 5	71 ± 9

Actiware sleep variables were statistically significant compared to all comparable WORK-A measures (^*^ represents significance at *P* ≤ 0.001, compared against each subsequent column)

### Comparing measures of sleep duration in ROTC cadets via Actiware sleep summary statistics and the WORK-A to self-report measures of sleep

**Fig. 2:**
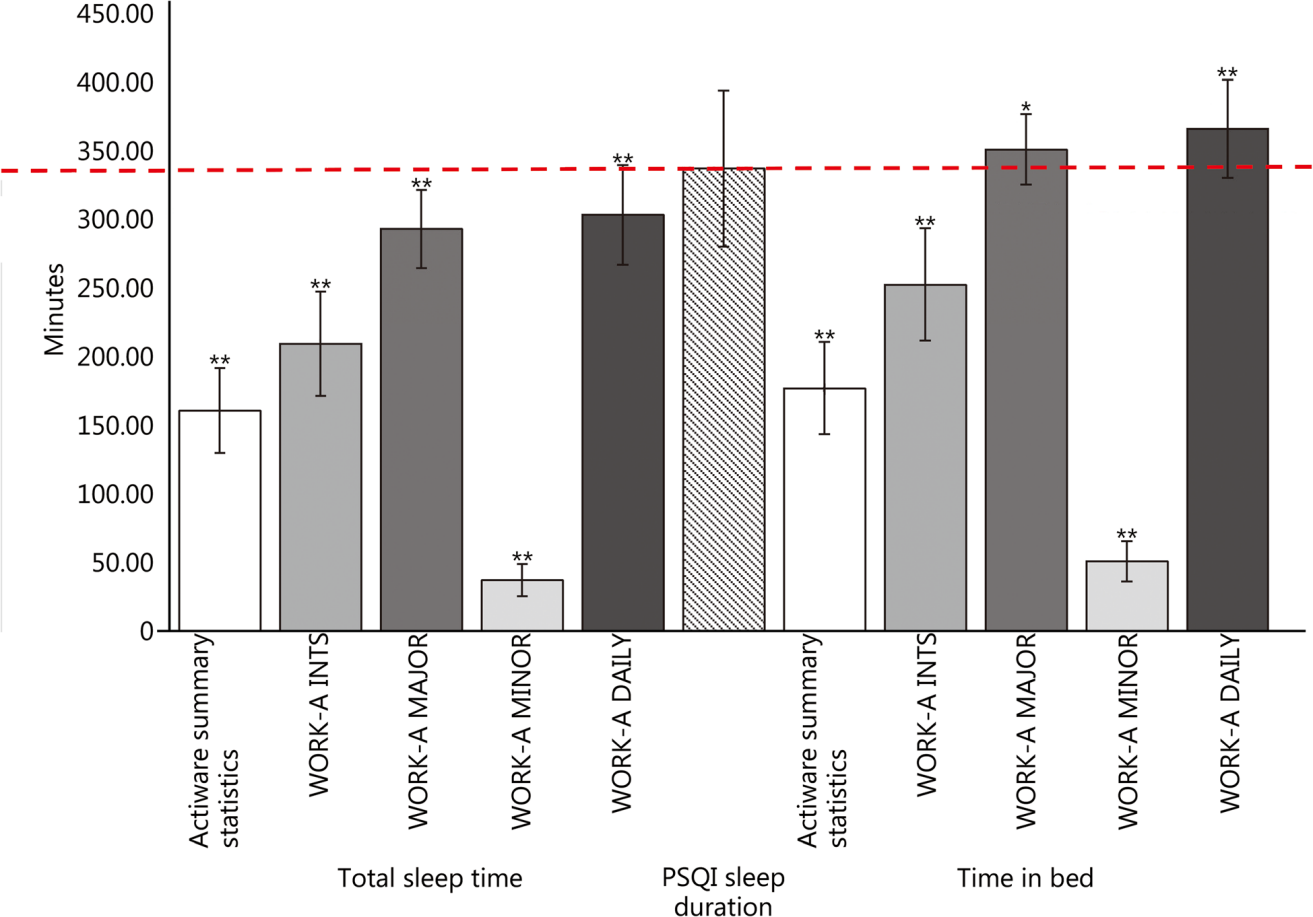
Average total sleep time and time in bed (in minutes) determined from actigraphy using Actiware versus WORK-A analysis compared to self-report sleep duration .Average TST and TIB as calculated by the Actiware sleep summary statistics (), WORK-A all intervals (), WORK-A major intervals only (), WORK-A minor intervals only () and WORK-A daily averages () in comparison to average self-report nightly sleep duration as measured by the Pittsburgh Sleep Quality Index (PSQI;) in minutes. The PSQI sleep duration is represented by the red dashed line (). * represents significance at *P* ≤ 0.05; ** represents significance at *P* ≤ 0.001

The results for sleep duration as measured by the PSQI compared to TST and TIB from Actiware summary statistics and the WORK-A are summarized in Fig. 2. Among the 286 ROTC Advanced Camp cadets who wore Actiwatches throughout Advanced Camp, 140 (107 men and 33 women) also completed the PSQI on the last day of camp to provide a self-report of habitual sleep during the training event. In response to the question “During the last month, how many hours of actual sleep did you get at night?”, cadets reported 5.62 ± 0.95 h (337 ± 57 min) per night. Measures of sleep duration from the PSQI, Actiware sleep summary statistics and WORK-A TIB and TST were all significantly different (Fig. 2; all *P* ≤ 0.05).

**Fig. 3:**
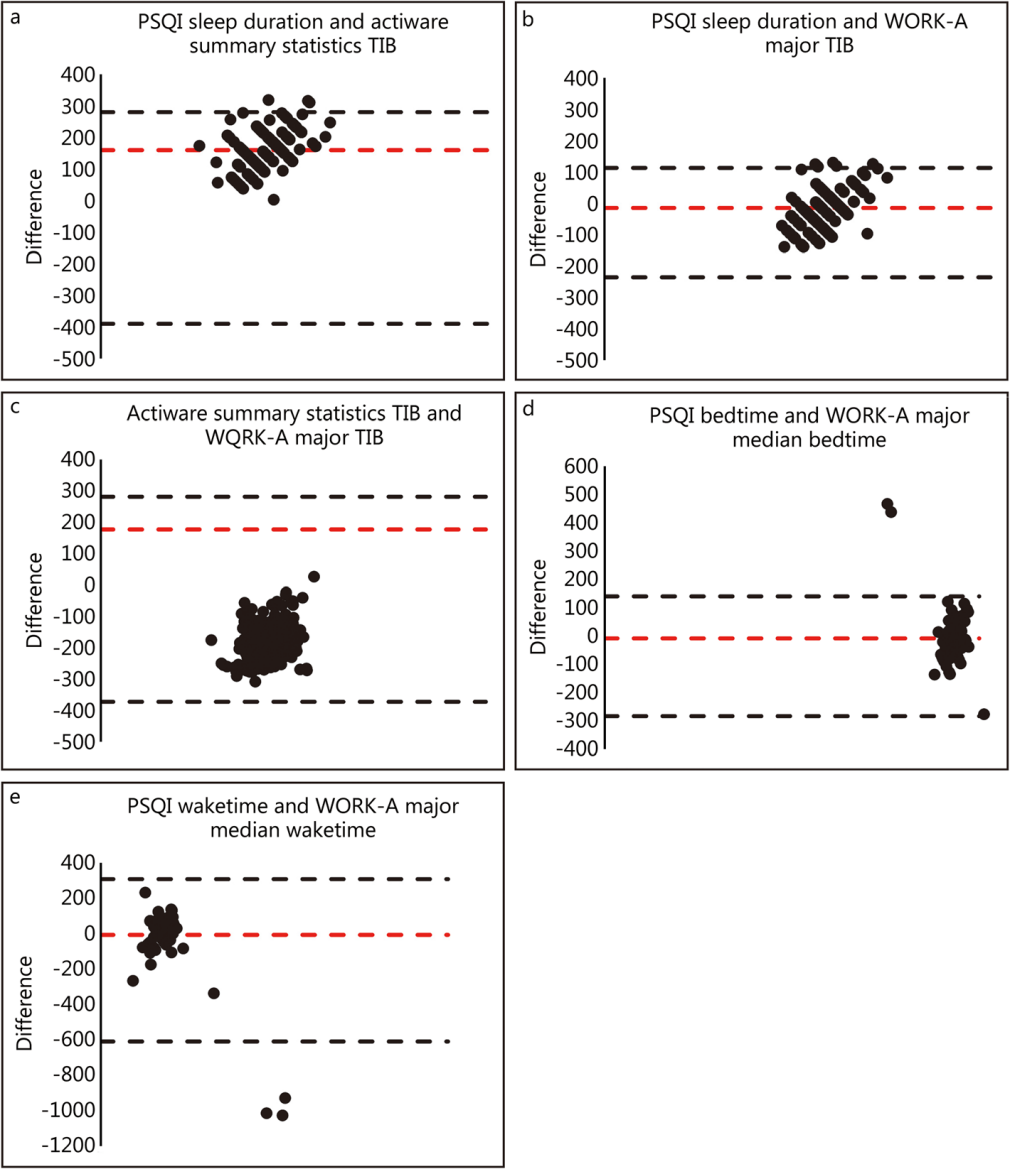
Bland-Altman plots comparing self-report and actigraphy measures of sleep. Bland-Altman plot of differences (*y*-axis) between self-report sleep (PSQI) and actigraphy measured sleep vs. the mean of the two measurements (*x*-axis). Bias is represented by the red dashed line (), while the upper and lower limits of agreement (LOA) are represented by the black dashed line (). **a**. Agreement between the PSQI sleep duration and Actiware summary statistics TIB (*P* ≤ 0.001). **b**. Agreement between the PSQI sleep duration and WORK-A major interval TIB (*P* = 0.006). **c**. Agreement between the Actiware summary statistics TIB and WORK-A major TIB (*P* ≤ 0.001). **d**. Agreement between the PSQI reported bedtime and WORK-A major interval median bedtime (*P* = 0.21). **e**. Agreement between the PSQI reported waketime and WORK-A major interval median waketime (*P* = 0.68)

Bland-Altman plots of the mean difference of TIB among Actiware data, WORK-A and PSQI are summarized in Fig. 3. One sample *t* test analysis revealed statistically significant differences, indicating a lack of agreement, between the PSQI sleep duration and both actigraphy-based measures of sleep duration (all *P* ≤ 0.05). Though still significantly different, the smallest mean difference was observed between the PSQI sleep duration and WORK-A major TIB (Fig. 3b; *P* = 0.006) compared to that between PSQI sleep duration and Actiware TIB (Fig. 3a; *P* ≤ 0.001) or Actiware TIB and WORK-A major TIB (Fig. 3c; *P* ≤ 0.001).

Cadets reported an average bedtime of 22:13 ± 0:40 h for the PSQI question “During the last month, when have you usually gone to bed?” and an average wake time of 04:31 ± 0:47 h. As depicted in Fig. 3d and e, the WORK-A major interval median bedtime (22:21 ± 1:30) and waketime (04:30 ± 2:17) were in agreement with PSQI-reported bedtime (*P* = 0.21) and waketime (*P* = 0.68), respectively. All other bedtime and wake time parameters were significantly different from those from the PSQI (all *P* ≤ 0.05).

### ROTC cadets failed to meet the minimum sleep duration

WORK-A daily TST showed an average of 303 ± 44 min of sleep per day. However, cadets failed to meet the specified minimum sleep duration (≥ 4 h / 24-h period [41]) for an average of 4 ± 3 days during wear time. Only 10% of the 286 cadets (*n* = 30) consistently met the minimum for all days that they wore the watch. Fifty-seven percent (57%; *n* = 162) of all cadets failed to meet the minimum for between 1 and 4 days, 10% of cadets (*n* = 50) failed to meet the minimum for between 5 and 10 days and 5% of all cadets (*n* = 16) failed to meet the minimum for more than 10 days during Advanced Camp.

## Discussion

### The WORK-A effectively profile soldier sleep compared to Actiware sleep summary statistics

The advantage of actigraphy as a research tool is its ability to measure sleep accurately in a real-world environment. This ability is particularly important in the operational context where sleep loss could increase the risk for accidents or mission-critical errors. While automated scoring algorithms for sleep-wake determination for actigraphy have been validated against PSG and self-report, much nuance can be lost through the averaging of these data. The result is a vague estimation of habitual sleep, which may inhibit researchers’ ability to interpret erratic sleep patterns, investigate the relationship between specific sleep behaviors and health or performance outcomes, or identify target behaviors for the improvement of sleep duration or quality.

The WORK-A provides researchers with an adaptable range of measures for understanding sleep that do not redefine actigraphy sleep-wake determination. Both Actiware sleep summary statistics and the WORK-A rely on the same set of rules for sleep-wake determination (i.e., those outlined by Philips Actiware). The WORK-A is not a novel computational method for the determination of sleep but a set of procedures for standardized processing of actigraphy data that conforms to Philips sleep-wake determination algorithms (as described in the Methods section) and a toolkit of additional variables that can be calculated from sleep-wake data to provide a more robust understanding of sleep in an operational context.

### The pre-processing procedures in the WORK-A may be more suitable for sleep measurements in service members

In the current study, there were differences in sleep measures due to the WORK-A pre-processing procedures (i.e., verifying that Actiware autoscoring follows sleep-wake determination according to the algorithms’ parameters). These cleaning procedures alone accounted for approximately an additional 53 min of sleep in TST, 83 min for TIB, 12 min from SOL and WASO, and a drop in SE from 82 to 79% compared to actigraphy data that were not processed by a technician. Technician logs for this dataset indicated that there were more instances where the autoscore function failed to catch sleep or wake onset criteria in the actogram than that of data loss including off-wrist inactivity. Therefore, the difference in key sleep variables between the summary statistics and the WORK-A could not be attributed to off-wrist or exclusionary periods. This discrepancy highlights a potential shortcoming in Philips software to reliably determine sleep-wake onset using actigraphy. It should be noted that while Philips Actiware and Actiwatch devices are validated for use in healthy adults, children and sleep-disordered individuals [20–22, 43], they were not designed for explicit use in the military operational domain. The pre-processing of actigraphy data to WORK-A standards is time- and labor-intensive, requiring a commitment of approximately 30 min per participant. Autoscoring would likely be sufficient for populations adhering to a more predictable schedule.

### Implications for sleep, health and performance management of ROTC cadets

Although significantly different, the WORK-A major interval measures of sleep came closer to agreement with the PSQI measures of sleep than did the Actiware summary statistics or other WORK-A interval statistics. However, all measures of sleep duration during camp were well below the National Sleep Foundation’s recommended 8–10 h for teenagers and 7–9 h for young adults [39]. The WORK-A additionally indicated that only 10% of cadets consistently met the specified minimum of 4 h of sleep/24-h period during camp. Insufficient sleep is problematic for the health and academic performance of college-aged students in general [44, 45], and it constitutes an additional risk to cadets. Insufficient sleep and poor quality sleep may not only negatively impact the immediate performance of ROTC cadets [46], but they can also affect decision-making [47], risk-taking behavior [48], emotion regulation [49] and constructive thinking [50]. It is reasonable to postulate that adapting to patterns of poor sleep behavior early on could set the stage for detrimental leadership throughout a cadet’s military career in a manner that not only affects them but also their future subordinates.

As visualized in Fig. 1, cadets slept mainly at night. However, the hourly analysis of sleep occurrence indicated a 1–5% likelihood of sleep occurrence during daytime hours, and the minor interval analysis indicated short periods of sleep (i.e., napping) that may not be otherwise have been captured by self-report (the PSQI only asks specifically about nighttime sleep) or traditional analysis of actigraphy data. It should be noted that the ROTC Advanced Camp is highly structured, thus constraining opportunities for sleep. Cadets are chaperoned by US Army soldiers, engage in training events throughout each day and adhere to a structured schedule. Sleep occurrences in other military populations are likely to be less predictable or consolidated than those of cadets. Napping has been shown to be related to a number of physical and mental health parameters as well as cognitive ability [51–55]. It is possible that taking advantage of brief opportunities for rest could be instrumental in increasing overall sleep duration and improving soldier readiness. Additionally, there are innumerable aspects of a military lifestyle, such as activity patterns [56], diet [57, 58], or work schedule [59], which may modulate the relationship between sleep and performance in service members differentially by individual factors such as gender, psychological distress, or chronotype [60–62]. Future analyses will investigate the relationship between sleep, performance and related factors in military groups across the operational domain.

### Limitations

One limitation to profiling sleep in the operational context is the lack of corroborating objective data. More detailed measures of sleep, such as daily sleep diaries, PSG, or direct observation, are often logistically infeasible to obtain given the mission-critical nature of the military domain. Measuring soldier sleep with the current limitations to data collection will, however, permit researchers to begin to model sleep patterns in the military domain, opening up the possibility of replicating these conditions in a controlled laboratory environment or closing the gap in our capacity to collect robust data through new technologies or procedures.

An important next step towards validating the WORK-A will be comparing the methodology against PSG in a controlled environment that replicates erratic sleep schedules. To achieve this difficult goal, we plan to collect actigraphy data concurrently with PSG during an in-lab multiple-day sleep restriction protocol. Modified maintenance of wakefulness tests (MWT), which discourage sleep but allow participants to remain asleep if sleep does occur, will be performed throughout the waking period. These conditions should mimic the need to stay vigilant despite insufficient sleep, which is characteristic of sustained military operations and serve as an appropriate proxy for testing the WORK-A in the laboratory.

ROTC cadets in the current study wore Actiwatches throughout Advanced Camp but only completed a one-time habitual self-report of their sleep behavior during Camp (i.e., PSQI). Moreover, the study design allowed participants to consent separately to wear the Actiwatch devices and to complete the surveys. While this design choice yielded greater participation in the actigraphy portion of the study than if cadets were required to complete all aspects of the study, comparisons to self-report are limited to the subset who agreed to participate in both self-reports of sleep and actigraphy. Not only can self-report be difficult to collect and ultimately subjective, but the PSQI was designed to estimate sleep quality, not to directly measure habitual sleep duration or bed- and waketimes. Future validation of the WORK-A pre-processing procedures for actigraphy will require a comparison against another daily measure of real-world sleep, such as the Pittsburgh Sleep Diary [63]. Moreover, the WORK-A has been demonstrated here in a population of ROTC cadets in a highly controlled environment. Adjustments to the WORK-A may be necessary as it is applied to soldier sleep data in less predictable mission conditions. Another limitation is Actiware sleep summary statistics’ inability to calculate average bed- and waketimes, which prevented the comparison of un-processed actigraphy bed- and waketimes to self-report times. This limitation highlights the need for additional computation of variables from actigraphy data, such as those provided by the WORK-A.

## Conclusions

In light of these limitations, the WORK-A represents a step forward towards understanding the role of sleep in soldier readiness. The intent is to apply the methods described above to actigraphy datasets collected from across a wide range of service member groups in simulated combat and deployment operations to investigate how sleep behavior may play a role in vigilance, decision-making, team-level performance, and a number of other military-relevant outcome variables. The WORK-A will also provide a baseline understanding of soldier sleep for the development of military-relevant laboratory sleep studies, provide guidance to military leadership and feedback to service members [64], and ultimately, better protect our nation’s soldiers by improving U.S. Army policies and doctrine regarding sleep behavior.

## Data Availability

The datasets generated and/or analyzed during the current study are not publicly available due to the sensitive nature of military training operations and ongoing data analysis but are available from the corresponding author on reasonable request.

## Abbreviations

AC: Activity count
DSI: Daily sleep intervals
MWT: Maintenance of wakefulness test
ORT: Operational research team
PSG: Polysomnography
PSQI: Pittsburgh Sleep Quality Index
RAR: Rest-activity rhythms
ROTC: Reserve Officer Training Corps
SE: Sleep efficiency
SOL: Sleep onset latency
SSBM: Soldier sleep below minimum
TIB: Time in bed
TST: Total sleep time
WASO: Wake after sleep onset
WORK-A: WRAIR Operational Research Kit-Actigraphy
WRAIR: Walter Reed Army Institute of Research
